# Supplementary material for: Challenges and advances for transcriptome assembly in non-model species
Source: PLoS One. 2017 Sep 20;12(9):e0185020. doi: 10.1371/journal.pone.0185020 (PMC5607178; doi:10.1371/journal.pone.0185020)
Supplement: S1 Table — (DOCX) [file pone.0185020.s001.docx]

## S1 table: output example of the transcriptome-guided assembly pipeline (simulations) corresponding to a collection of database entries displaying the number of identified reads for each gene-id.

| **gene_id** | **gene_name** | **transcript_biotype** | **gene_length** | **transcript_length_Min** | **transcript_length_Max** |  |
| --- | --- | --- | --- | --- | --- | --- |
| ENSDARG00000037978 | clec19a | protein_coding | 3823 | 3823 | 3823 |  |
| ENSDARG00000038094 | clec16a | protein_coding | 6533 | 6533 | 6533 |  |
| ENSDARG00000089402 | MAP7D1 | protein_coding | 934 | 934 | 934 |  |
| ENSDARG00000077475 | RBBP6 | protein_coding | 984 | 984 | 984 |  |
|  |  |  |  |  |  |  |
|  |  |  |  |  |  |  |
|  |  |  |  |  |  |  |
| **gene_id** | **gene_start_position** | **gene_end_position** | **AvgHSP** | **AvgRead** | **AvgCov** | **chromosome** |
| ENSDARG00000037978 | 31030449 | 31059886 | 100 | 100 | 1,02 | 3 |
| ENSDARG00000038094 | 26692917 | 26775785 | 100 | 100 | 0,796 | 3 |
| ENSDARG00000089402 | 3959680 | 3965688 | 189 | 200 | 5,996 | 19 |
| ENSDARG00000077475 | 12646228 | 12648095 | 194,103 | 200 | 13,821 | 11 |
|  |  |  |  |  |  |  |
|  |  |  |  |  |  |  |
|  |  |  |  |  |  |  |
|  |  |  |  |  |  |  |
| **gene_id** | **Simulated reads** | **Correctely assigned reads** | **Total assigned reads** | **reads from other genes** | **Hit_multigene** |  |
| ENSDARG00000037978 | 39 | 39 | 39 | 0 | 0 |  |
| ENSDARG00000038094 | 66 | 52 | 52 | 0 | 27 |  |
| ENSDARG00000089402 | 47 | 13 | 28 | 15 | 47 |  |
| ENSDARG00000077475 | 50 | 26 | 68 | 42 | 29 |  |

### Table legend: each line corresponded to a gene and presented:

gene_id: identification of the gene

gene_name: name of the gene

Transcript_biotype: function of the transcript

Gene_length: length of the coding gene

transcript_length_Min: length of the shortest transcript

transcript_length_Max: length of the longest transcript

gene_start_position: first position of the gene on its chromosome

gene_end_position: last position of the gene on its chromosome

AvgHSP: mean length of the HSPs for this gene

AvgRead : mean length of the reads for this gene

AvgCov: mean coverage corresponding to the total assigned reads x AvgRead / Gene_length

Chromosome: chromosome numbering

Simulated reads: number of generated reads for a gene

Correctly assigned reads: number of generated reads coming from this gene

Total assigned reads: number of generated reads assigned to this gene

reads from other genes: number of generated reads from another gene assigned to this gene

Hit_multigene: number of generated reads assigned to multiple genes with identical score

A complete information for each parameter is found at <http://www.ensembl.org/Help/Faq?id=468>.
