# Supplementary material for: Challenges and advances for transcriptome assembly in non-model species
Source: PLoS One. 2017 Sep 20;12(9):e0185020. doi: 10.1371/journal.pone.0185020 (PMC5607178; doi:10.1371/journal.pone.0185020)

S2 Table: Prediction of true affectation probability for recovery rate and specificity rate

1. Confidence intervals of odds-ratio (OR) for the probability of the recovery rate *rr* (i.e. a read simulated from a gene-id correctly assigned to this gene-id) as a function of model terms. The *rr* is modeled using a mixed logistic model that incorporates gene length as a quantitative variable, and fixed factors for divergence (0, 5, 15 and 30%), read length (100, 150, 200 and 350 bases) and all two-way interactions.

|  | **OR Estimation** | **Lower Limit** | **Upper Limit** |
| --- | --- | --- | --- |
| Intercept | 9.99730 | 9.61275 | 10.39723 |
| divergence5 | 0.91602 | 0.90859 | 0.92351 |
| divergence15 | 0.72723 | 0.72146 | 0.73304 |
| divergence30 | 0.03950 | 0.03924 | 0.03977 |
| reads_length150 | 1.61811 | 1.60748 | 1.62880 |
| reads_length200 | 2.37415 | 2.35340 | 2.39508 |
| reads_length350 | 4.79917 | 4.75099 | 4.84784 |
| gene_length | 1.00036 | 1.00034 | 1.00037 |
| divergence5:reads_length150 | 1.01327 | 1.00445 | 1.02216 |
| divergence15:reads_length150 | 1.07127 | 1.06215 | 1.08046 |
| divergence30:reads_length150 | 0.87261 | 0.86679 | 0.87847 |
| divergence5:reads_length200 | 0.99084 | 0.97931 | 1.00251 |
| divergence15:reads_length200 | 1.04001 | 1.02817 | 1.05199 |
| divergence30:reads_length200 | 0.68376 | 0.67770 | 0.68987 |
| divergence5:reads_length350 | 1.01705 | 1.00331 | 1.03097 |
| divergence15:reads_length350 | 1.12719 | 1.11221 | 1.14237 |
| divergence30:reads_length350 | 0.38642 | 0.38249 | 0.39038 |
| divergence5:gene_length | 1.00001 | 1.00001 | 1.00001 |
| divergence15:gene_length | 1.00002 | 1.00002 | 1.00002 |
| divergence30:gene_length | 0.99989 | 0.99989 | 0.99989 |
| reads_length150:gene_length | 0.99998 | 0.99998 | 0.99998 |
| reads_length200:gene_length | 0.99997 | 0.99997 | 0.99997 |
| reads_length350:gene_length | 0.99995 | 0.99995 | 0.99995 |

1. Analysis of deviance table evaluating the model of *rr*.

|  | **Df** | **Chisq** | **P** |
| --- | --- | --- | --- |
| divergence | 3 | 1728134.9 | < 2.2e-16 |
| reads_length | 3 | 109548.1 | < 2.2e-16 |
| gene_length | 1 | 2586.4 | < 2.2e-16 |
| divergence:reads_length | 9 | 100377.4 | < 2.2e-16 |
| divergence:gene_length | 3 | 51745.9 | < 2.2e-16 |
| reads_length:gene_length | 3 | 11663.2 | < 2.2e-16 |

1. Confidence intervals of odds-ratio (OR) for the probability of the specificity rate *sr* (i.e. a read assigned to a gene-id belonging to this gene-id). The specificity rate is modeled using a mixed logistic model that incorporates gene length as a quantitative variable, and fixed factors for divergence (0, 5, 15 and 30%) and read length (100, 150, 200 and 350 bases).

|  | **OR Estimation** | **Lower Limit** | **Upper Limit** |
| --- | --- | --- | --- |
| Intercept | 137740.93741 | 123712.13295 | 153360.59113 |
| divergence5 | 0.04749 | 0.04476 | 0.05038 |
| divergence15 | 0.01416 | 0.01336 | 0.01501 |
| divergence30 | 0.00317 | 0.00299 | 0.00336 |
| reads_length150 | 2.36501 | 2.33912 | 2.39118 |
| reads_length200 | 3.73182 | 3.67558 | 3.78893 |
| reads_length350 | 7.39697 | 7.26656 | 7.52971 |
| gene_length | 1.00024 | 1.00021 | 1.00027 |

1. Analysis of deviance table for the model of specificity rate.

|  | **Df** | **Chisq** | **P** |
| --- | --- | --- | --- |
| divergence | 3 | 176877.16 | < 2.2e-16 |
| reads_length | 3 | 68777.74 | < 2.2e-16 |
| gene_length | 1 | 265.99 | < 2.2e-16 |

1. Predicted specificity rate (*sr*) using the mixed logistic modelization as a function gene length, read length and divergence between target and reference transcriptomes: red lines denote 0% divergence; green 5%; blue 15% & purple 30%. Solid lines correspond to the median of predictions, conditioned on random variation among genes, with 80% prediction intervals indicated by dashed lines. Read length increases downward: 100bp reads (A); 150 bases (B); 200 bases (C); 350 bases (D).


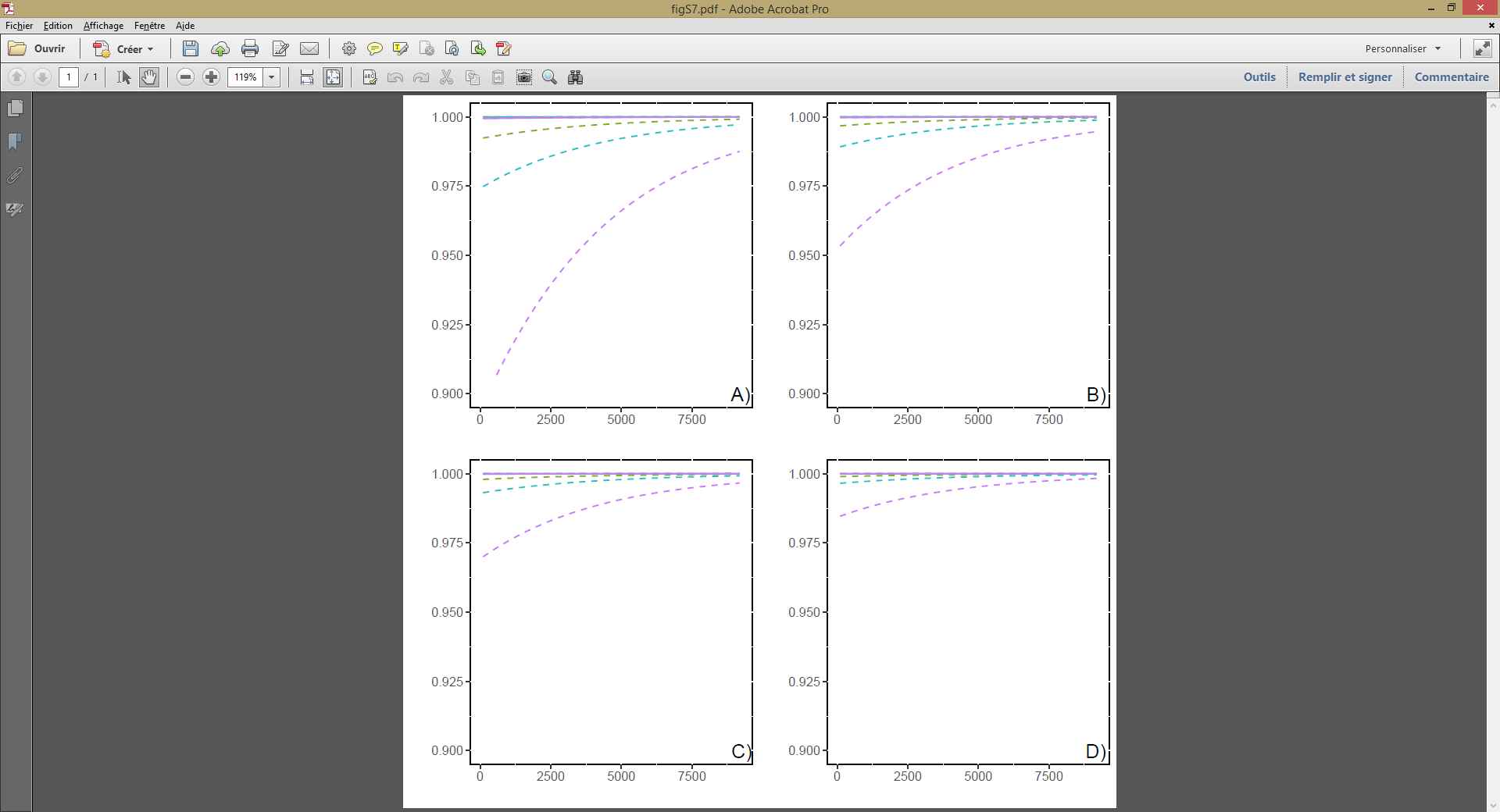

Supplement: S2 Table — (DOCX) [file pone.0185020.s002.docx]
