# Supplementary material for: Challenges and advances for transcriptome assembly in non-model species
Source: PLoS One. 2017 Sep 20;12(9):e0185020. doi: 10.1371/journal.pone.0185020 (PMC5607178; doi:10.1371/journal.pone.0185020)
Supplement: S3 Table — (DOCX) [file pone.0185020.s003.docx]

S3 table: Classification of simulated reads assigned to the *D. rerio* transcriptome using blastn, based on the five categories defined in the manuscript (mixed, donor, recipient, perfect, undetectable). A - number (left table) and percentage (right table) of identified genes for the five different categories.

|  | 10X | **100** | **150** | **200** | **350** |  | **100** | **150** | **200** | **350** |
| --- | --- | --- | --- | --- | --- | --- | --- | --- | --- | --- |
|  | divergence=0 |  |  |  |  |  |  |  |  |  |
| mixed | rr<1,sr<1 | 0 | 44 | 0 | 251 |  | 0.00 | 0.14 | 0.00 | 0.79 |
| donor | rr<1,sr=1 | 8368 | 5932 | 4491 | 2743 |  | 26.20 | 18.57 | 14.06 | 8.59 |
| recipient | rr=1,sr<1 | 0 | 4 | 0 | 38 |  | 0.00 | 0.01 | 0.00 | 0.12 |
| perfect | rr=1,sr=1 | 21913 | 24320 | 25820 | 27289 |  | 68.60 | 76.13 | 80.83 | 85.43 |
| undetectable | rr=0,sr=N.A. | 1663 | 1644 | 1633 | 1623 |  | 5.21 | 5.15 | 5.11 | 5.08 |
|  | Total | 31944 | 31944 | 31944 | 31944 |  | 100 | 100 | 100 | 100 |
|  | divergence=5 |  |  |  |  |  |  |  |  |  |
| mixed | rr<1,sr<1 | 1494 | 969 | 542 | 464 |  | 4.68 | 3.03 | 1.70 | 1.45 |
| donor | rr<1,sr=1 | 6929 | 5036 | 4016 | 2510 |  | 21.69 | 15.77 | 12.57 | 7.86 |
| recipient | rr=1,sr<1 | 156 | 98 | 66 | 107 |  | 0.49 | 0.31 | 0.21 | 0.33 |
| perfect | rr=1,sr=1 | 21687 | 24186 | 25671 | 27222 |  | 67.89 | 75.71 | 80.36 | 85.22 |
| undetectable | rr=0,sr=N.A. | 1678 | 1655 | 1649 | 1641 |  | 5.25 | 5.18 | 5.16 | 5.14 |
|  | Total | 31944 | 31944 | 31944 | 31944 |  | 100 | 100 | 100 | 100 |
|  | divergence=15 | |  |  |  |  |  |  |  |  |
| mixed | rr<1,sr<1 | 3382 | 1981 | 1244 | 738 |  | 10.59 | 6.20 | 3.89 | 2.31 |
| donor | rr<1,sr=1 | 9571 | 4558 | 3539 | 2267 |  | 29.96 | 14.27 | 11.08 | 7.10 |
| recipient | rr=1,sr<1 | 391 | 178 | 128 | 158 |  | 1.22 | 0.56 | 0.40 | 0.49 |
| perfect | rr=1,sr=1 | 16864 | 23497 | 25307 | 27070 |  | 52.79 | 73.56 | 79.22 | 84.74 |
| undetectable | rr=0,sr=N.A. | 1736 | 1730 | 1726 | 1711 |  | 5.43 | 5.42 | 5.40 | 5.36 |
|  | Total | 31944 | 31944 | 31944 | 31944 |  | 100 | 100 | 100 | 100 |
|  | divergence=30 | |  |  |  |  |  |  |  |  |
| mixed | rr<1,sr<1 | 7370 | 3647 | 2248 | 1214 |  | 23.07 | 11.42 | 7.04 | 3.80 |
| donor | rr<1,sr=1 | 21481 | 24715 | 25725 | 25888 |  | 67.25 | 77.37 | 80.53 | 81.04 |
| recipient | rr=1,sr<1 | 76 | 71 | 77 | 76 |  | 0.24 | 0.22 | 0.24 | 0.24 |
| perfect | rr=1,sr=1 | 700 | 1184 | 1544 | 2401 |  | 2.19 | 3.71 | 4.83 | 7.52 |
| undetectable | rr=0,sr=N.A. | 2317 | 2327 | 2350 | 2365 |  | 7.25 | 7.28 | 7.36 | 7.40 |
|  | Total | 31944 | 31944 | 31944 | 31944 |  | 100 | 100 | 100 | 100 |

B-Proportion of gene types recovered in divergent simulations by size-class of gene. 100, 150, 200, 350 base reads are plotted with 0%, 5%, 15% and 30% divergence. Gene types are described in the legend.
