# Supplementary material for: Challenges and advances for transcriptome assembly in non-model species
Source: PLoS One. 2017 Sep 20;12(9):e0185020. doi: 10.1371/journal.pone.0185020 (PMC5607178; doi:10.1371/journal.pone.0185020)
Supplement: S1 Protocol — (DOCX) [file pone.0185020.s004.docx]

## S1 protocol: Simulating reads for efficiency and performance testing

The read length variable is used to represent the classical range of Illumina sequencing lengths available, irrespective of whether a single-end or paired-end strategy is used. Simulated reads of length *l* (100, 150, 200 and 350 bases) are sampled from the longest transcript (of length *L*) for each identified gene, taking one iteration *i* from a Uniform distribution U(1,*L*). Each transcript is sampled at exactly 10X coverage (*L*/*l*=10). The position *i* defines the starting position of the read and the position *i*+*l*-1 defines the ending position of the read. When *l*>*L* (the simulated read would be longer than the transcript), the read takes *L* as its length. For the transcriptome of *D. rerio*, among the 31,953 genes annotated, nine have a length < 30 bases and are discarded from the simulation, 809 have a length ranging from 30 to 100 bases, 2,051 have a length ranging from 100 to 149, 452 have a length ranging from 150 to 199, 648 have a length ranging from 200 to 349, and 27,993 have a length ≥ 350 bases.

Sequence divergence is used to simulate confounding processes such as sequence error rate, polymorphism, and species divergence. We mimic sequence divergence (considering four values of divergence at level *b*: 0%, 5%, 15% and 30%) generating *N* mutations sampled from a Uniform distribution U(1,*L*) where *L* is the length of the longest transcript of the gene and  *N* = *b* (sequence divergence in %) * *L* (length of the transcript).

The combination of four conditions for the read length and four conditions for the sequence divergences provides 16 sets of simulated reads.
