# Supplementary material for: Challenges and advances for transcriptome assembly in non-model species
Source: PLoS One. 2017 Sep 20;12(9):e0185020. doi: 10.1371/journal.pone.0185020 (PMC5607178; doi:10.1371/journal.pone.0185020)
Supplement: S2 Protocol — (DOCX) [file pone.0185020.s005.docx]

## S2 Protocol: Illumina library production

The TruSeq Stranded mRNA Library Preparation kit (Illumina Inc., USA) was used according to the manufacturer's protocol with the following modifications. In brief, poly-A containing mRNA molecules were purified from 2 µg of total RNA using poly-T oligo attached magnetic beads. The purified mRNA was fragmented by thermal shearing in fragmentation buffer heated to 94°C in a thermocycler for 4 min. A fragmentation time of 4 min was used to yield library fragments of 250-500 bases. First strand cDNA was synthesized using random primers to eliminate the general bias towards the 3' end of the transcript. Second strand cDNA synthesis, end repair, A-tailing, and adapter ligation was done in accordance with the protocols supplied by the manufacturer. Purified cDNA templates were enriched by 12 cycles of PCR for 10 s at 98°C, 30 s at 65°C, and 30 s at 72°C using PE1.0 and PE2.0 primers and with Phusion DNA polymerase. Each indexed cDNA library was verified using a DNA 1000 Chip on a Bioanalyzer 2100, quantified by real time PCR with the KAPA Library Quantification Kit for Illumina Sequencing Platforms (Kapa Biosystems Ltd, SA), and adjusted to 4 nM in water.

The final pooled cDNA library was sequenced using the Illumina paired-end protocol on a Miseq sequencer (AGAP, Montpellier, France), for 2x 250 cycles. Libraries were diluted to 2 nM with NaOH and 2.5 μL transferred into 497.5 μL HT1 to produce a final concentration of 10 pM. Illumina basecalling files were processed directly on the Miseq sequencer, and produced paired-end read files containing reads for each sample in Illumina 1.8 FASTQ format
