# Supplementary material for: Challenges and advances for transcriptome assembly in non-model species
Source: PLoS One. 2017 Sep 20;12(9):e0185020. doi: 10.1371/journal.pone.0185020 (PMC5607178; doi:10.1371/journal.pone.0185020)
Supplement: S1 Text — (DOCX) [file pone.0185020.s006.docx]

## S1 Text: Statistical framework for evaluating reads assignation performance

Let us define the population of reads having a length equal to l simulated from a transcript of a gene *g* of length *L* with divergence from the reference transcriptome at a level *b*. Let us define the following random variable *Y*=1 if the read truly belongs to this transcript, and 0 if not. Then the recovery rate *rr* will be defined as *rr=P(Y=1|l=l,b=b,L=L,g)*, the probability for a read simulated from a *gene-id* and correctly assigned to this *gene-id*.

We study the relationship of *l*, *L* and *b* with *rr* using the following mixed logistic model, *logit(P(Y=1|l=l,b=b,L=L,g)=α+β l + γ L + δ b +(βγ) l x L+(βδ ) lxb  +(γδ ) Lxb + G*, where α,  β,  γ and δ are parameters related to the fixed effects: length of reads *l* (considered as qualitative);  divergence *b* (qualitative), and; length of transcript *L* (quantitative). The terms (βγ), (βδ) and (γδ) refer to interactions between parameters, and *G* denotes a random variable following a centered Gaussian distribution of variance σG.

The model is fitted using R (version 3.3.1 [(R Core Team 2013)](https://paperpile.com/c/TXrMOS/rOxY) and the glmer function of the lme4 package. From the fixed effect estimates, confidence intervals of odds-ratio (OR) are built for interpretation. *In fine*, we obtained 4 * 4 factor combinations.

Let us define the population of reads having a length equal to *l* assigned to a transcript of a gene *g* of length *L* with a sequence divergence at a level *b*. Let us define the following random variable *Y*=1 if the read is truly generated from the transcript and 0 if not. Then the specificity rate (sr) will be defined as *sr=P(Y=1|l=l,b=b,L=L,g)*, that is the probability that a read simulated from a gene is found among reads assigned to this gene. Due to lack of model convergence when attempting to fit a similarly parameterized model as for the recovery rate, a simpler model (i.e. no interaction terms) is used to study *sr*. We study the relationship between *sr* and *l*, *L* and *b* using the following mixed logistic model, *logit(P(Y=1/l=l,b=b,L=L,g)=α+β l + γ L + δ b + G*,  where  α,  β,  γ and δ are parameters related to the fixed effects: length of reads *l* (considered as qualitative); length of transcript *L* (quantitative), and; sequence divergence *b* (qualitative). The term *G* refers to a random variable following a centered Gaussian distribution of variance σG. The model was fitted using R (version 3.3.1 [(R Core Team 2013)](https://paperpile.com/c/TXrMOS/rOxY) and the glmer function of the lme4 package [(Bates *et al.* 2014)](https://paperpile.com/c/TXrMOS/lcF2). From the fixed effect estimates, confidence intervals of odds-ratio (OR) were built for interpretation. *In fine*, we obtained 4 x 4 factor combinations.
