# Supplementary material for: Challenges and advances for transcriptome assembly in non-model species
Source: PLoS One. 2017 Sep 20;12(9):e0185020. doi: 10.1371/journal.pone.0185020 (PMC5607178; doi:10.1371/journal.pone.0185020)
Supplement: S2 Text — (DOCX) [file pone.0185020.s007.docx]

S2 Text: Computing resources and computing time

We used a computer with Linux OS (Debian 8 3.16.7-ckt25-1 x86_64 GNU/Linux) with an Intel Xeon E3-1245 V2 processor (Ivy Bridge generation, four cores with hyperthreading, 3.4 Ghz) and 32Gb of RAM.

Example from 6,748,779 150 base reads with 50 base overlap between read pairs and 0% divergence from the reference.

The merging step with PEAR 0.9.6 requires 163 s for 6,748,779 million sequences, (41,403 reads per second). The filtering step using custom scripts required 1,074 s (6,283 reads per second). The reads assignation step with blastn and custom scripts required 126,060 s (1 day, 11:02:23; 53 reads per second). The assembly step using Spades (v3.6.2) and/or CAP3 (v.021015) required 82,852 s (23h) for 30,623 genes (0.37 genes per second) with the dataset split between two threads. The contig assignation step using blastn and custom scripts required 8,642 s (2.4h) for 62,229 contigs (7.2 contigs per second).
