# Supplementary material for: Challenges and advances for transcriptome assembly in non-model species
Source: PLoS One. 2017 Sep 20;12(9):e0185020. doi: 10.1371/journal.pone.0185020 (PMC5607178; doi:10.1371/journal.pone.0185020)
Supplement: S1 Fig — (DOCX) [file pone.0185020.s008.docx]

S1 Fig: Nonparametric estimation of the contiguity (x-axis) and completeness scores (y-axis)

The distribution is obtained from *de* *novo* and guided-assembly pipelines. Colors increasing from yellow to dark red denote increasing gene densities. Note also that for clarity of visualization, only the non-perfect fraction of genes is displayed. At 5% divergence with 100bp reads assembled *de novo* (A) and with guided assembly (B); 200bp reads *de novo* (C) and guided assembly (D). The proportion of non-perfect genes is indicated at the top of each panel.

At 15% divergence with 100bp reads assembled de novo (E) and with guided assembly (F); 200bp reads *de novo* (G) and guided assembly (H). The proportion of non-perfect genes is indicated at the top of each panel.

At 30% divergence with 100bp reads assembled de novo (I) and with guided assembly (J); 200bp reads *de novo* (K) and guided assembly (L). The proportion of non-perfect genes is indicated at the top of each panel.
