# Supplementary material for: Challenges and advances for transcriptome assembly in non-model species
Source: PLoS One. 2017 Sep 20;12(9):e0185020. doi: 10.1371/journal.pone.0185020 (PMC5607178; doi:10.1371/journal.pone.0185020)

S2 Fig: Boxplots of contiguity and completeness

A- Boxplots of contiguity and completeness scores of RNA-seq data for *P. toxostoma* comparing the two assembly approaches *(de novo* and guided assembly).

B- Boxplots of contiguity and completeness scores of RNA-seq data for *Q. pubescens* comparing the two assembly approaches *(de novo* and guided assembly).


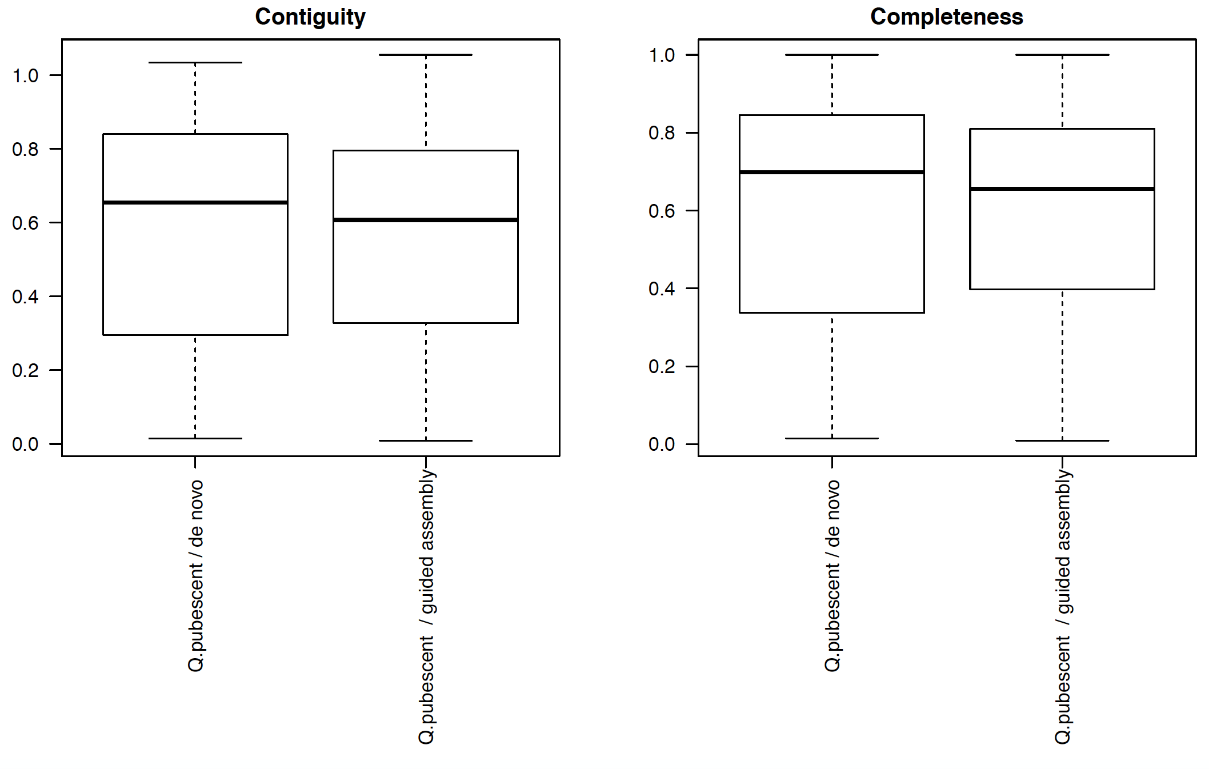

Supplement: S2 Fig — (DOCX) [file pone.0185020.s009.docx]
