# Supplementary material for: Challenges and advances for transcriptome assembly in non-model species
Source: PLoS One. 2017 Sep 20;12(9):e0185020. doi: 10.1371/journal.pone.0185020 (PMC5607178; doi:10.1371/journal.pone.0185020)

S3 Fig: Biological processes of the identified genes

A- We analyzed the inferred list of genes identified from the guided assembly of *P. toxostoma* using Panther version 10 [[48]](https://paperpile.com/c/C9bxu5/CkQzd). We found 24,241 assignments to GO biological processes categories (a gene may be assigned to multiple biological processes), 12,188 assignments to GO molecular function categories and 9,355 to GO cellular component categories. When comparing these results with the 31,953 genes from *D. rerio*, we found significant differences in the represented biological processes and molecular function categories (respectively *X^2^*=81.13, P=1.75 10^-11^ and *X^2^*=100.35, P=3.62 10^-18^.). Considering biological processes, the difference is due to the biological regulation category; for molecular function, it was due to receptor activity and signal transducer activity categories. Otherwise, the cellular component categories displayed no significant difference between the two species (*X^2^*=10.31, P=0.17).

Repartition of biological processes for the guided assembly describing the identified genes for the *P. toxostoma* assembly.

B- We analyzed the inferred list of genes identified from the guided assembly obtained for *Q. pubescens* using Panther version 10 [[48]](https://paperpile.com/c/C9bxu5/CkQzd). We found 13,776 assignments to GO biological processes categories (a gene may be assigned to multiple biological processes), 9,928 assignments to GO molecular function categories and 6,813 to GO cellular component categories. When comparing these results with the 29,971 genes from *V. vinifera*, we found no significant differences in enrichment sets between the two species (>0.01).

Repartition of biological processes for the guided assembly describing the identified genes for the *Q. pubescens* assembly.


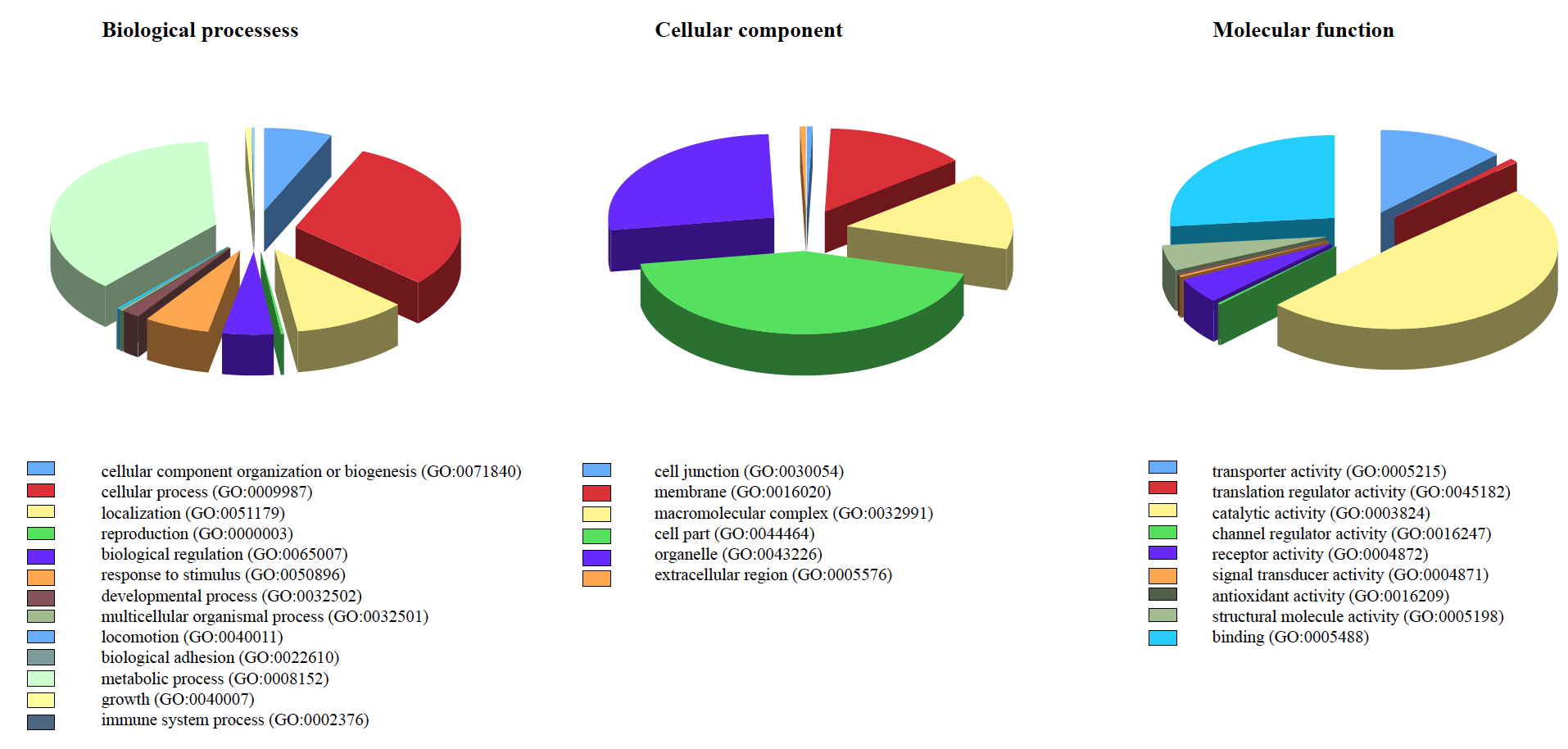

Supplement: S3 Fig — (DOCX) [file pone.0185020.s010.docx]
