# Supplementary material for: Challenges and advances for transcriptome assembly in non-model species
Source: PLoS One. 2017 Sep 20;12(9):e0185020. doi: 10.1371/journal.pone.0185020 (PMC5607178; doi:10.1371/journal.pone.0185020)
Supplement: S4 Fig — (DOCX) [file pone.0185020.s011.docx]

S4 Fig: Genetic divergence between genes from reference and non-model organisms.

Distribution of the genetic divergence between *Parachondrostoma toxostoma* and *Danio rerio* for the 18,519 orthologous genes identified. The x-axis displays the p-distance and the y-axis displays the density of genes observed. 5.90% of the genes display a divergence greater than 0.3 (red line), and 35.66% greater than 0.2 (orange line).


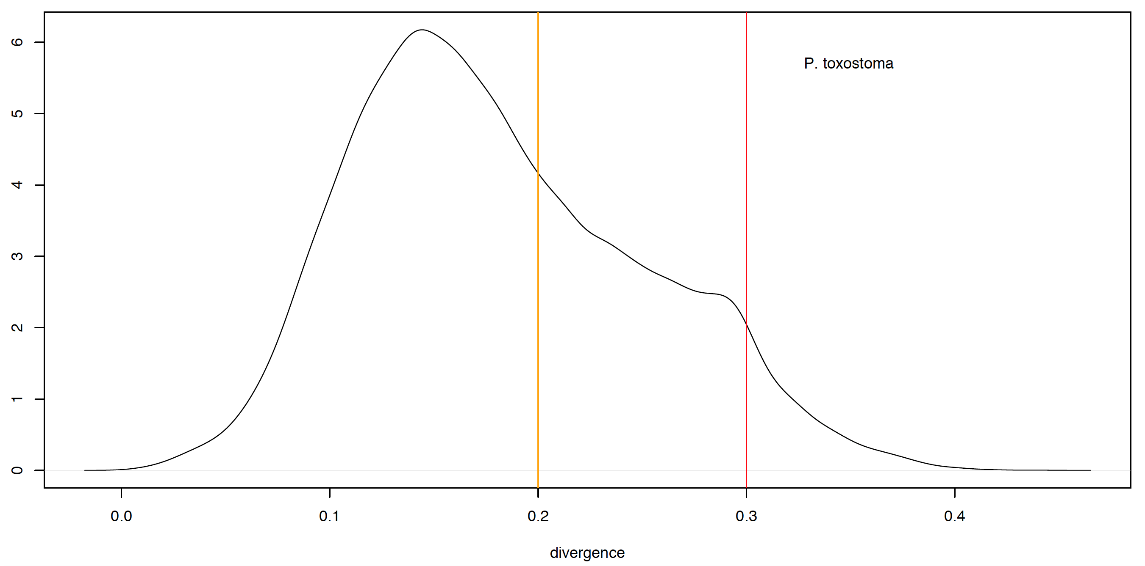


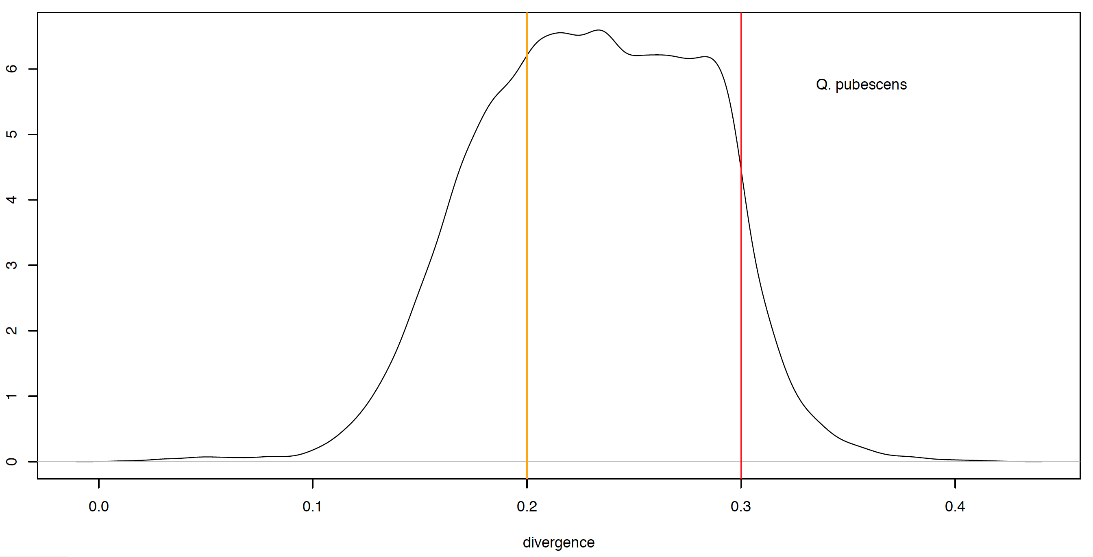
Distribution of the genetic divergence between *Quercus pubescens* and *Vitis vinifera* for the 16,326 orthologous genes identified. The x-axis displays the p-distance and the y-axis displays the density of genes observed. 7.74% display a divergence greater than 0.3 (red line) and 70.54% greater than 0.2(orange line).
