# Supplementary material for: Challenges and advances for transcriptome assembly in non-model species
Source: PLoS One. 2017 Sep 20;12(9):e0185020. doi: 10.1371/journal.pone.0185020 (PMC5607178; doi:10.1371/journal.pone.0185020)

S5 Fig: Interaction between coverage, gene size classes and completeness for *Parachondrostoma toxostoma* and *Quercus pubescens*

A- Interaction between gene length (x-axis) and coverage (y-axis). Red lines denote 10x coverage. Boxplots indicate the variation in coverage for genes belonging to different size classes (e.g. 119 corresponds to all genes ranging from 30 to 119 bases; 783 corresponds to all genes ranging from 120 to 783 bases) – note that 30 bases is the minimum size for a read to be considered. *P. toxostoma* data are plotted on the left, and *Q. pubescens* on the right.

B- Interaction between gene length (x-axis) and completeness (y-axis). A completeness equal to 1 indicates that the complete length of the longest transcript for the model organism (*D. rerio* or *V. vinifera*) was covered with contigs from the non-model organisms *P. toxostoma* (left) or *Q. pubescens* (right). Boxplots describe variation in completeness for genes belonging to the same size classes as in (A).

C- Interaction between depth of coverage (x-axis) and completeness (y-axis) for *P. toxostoma* (left) and *Q. pubescens* (right). Boxplots describe variation in completeness for the genes belonging to a range of coverage classes (e.g. 0.04corresponds to all genes presenting a coverage lower than 0.04x; 0.79 to all genes with a coverage from 0.04 to 0.79x). Box plot in green denotes the coverage class for the 10x coverage.

D- Interaction between the divergence as defined in S11 (x-axis) and completeness (y-axis) for *P. toxostoma* (left) and *Q. pubescens* (right).

A


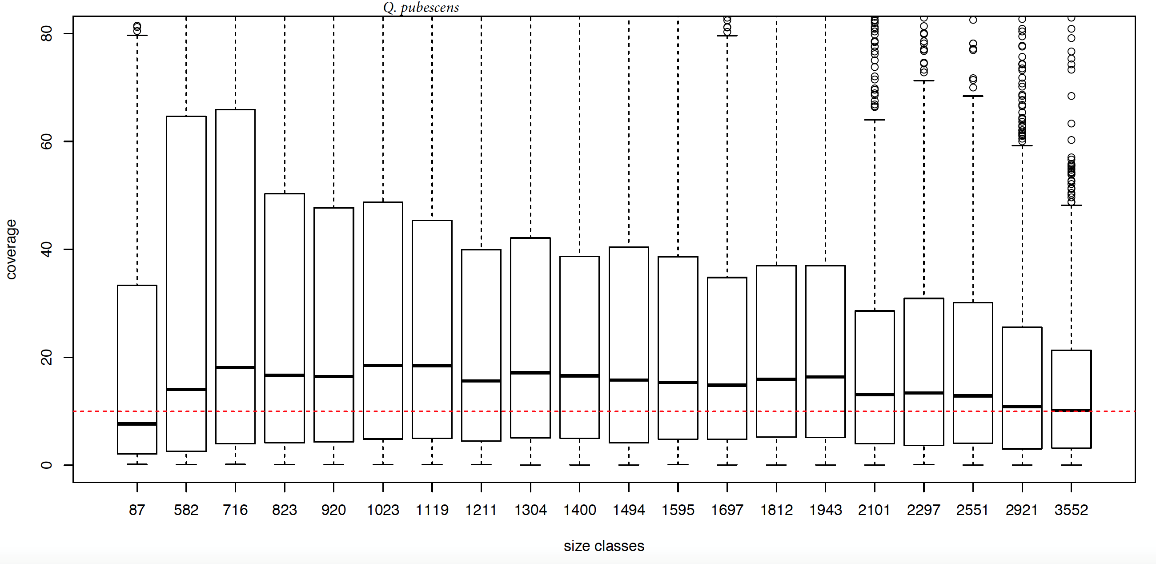

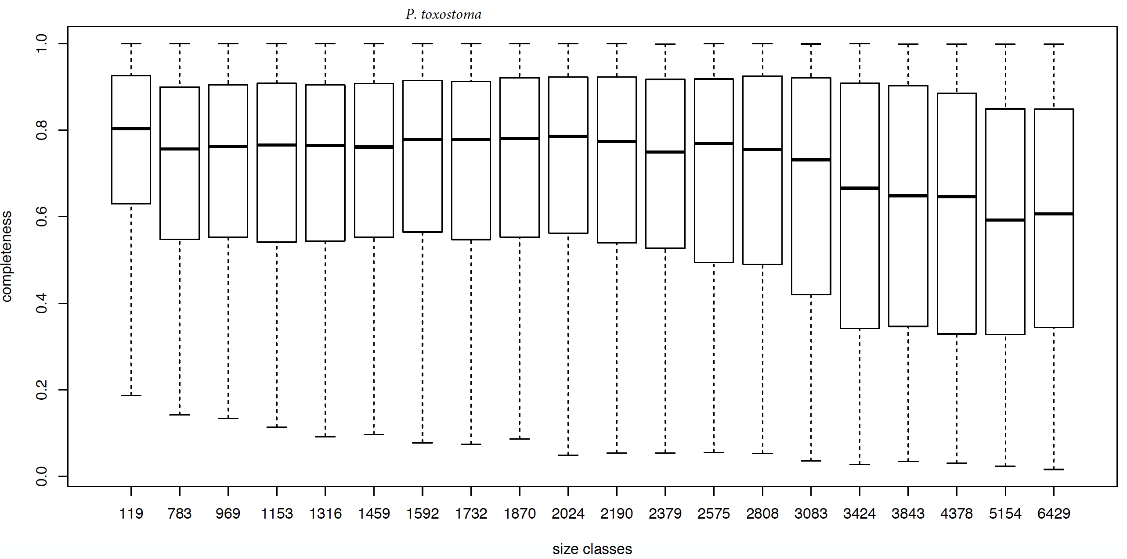

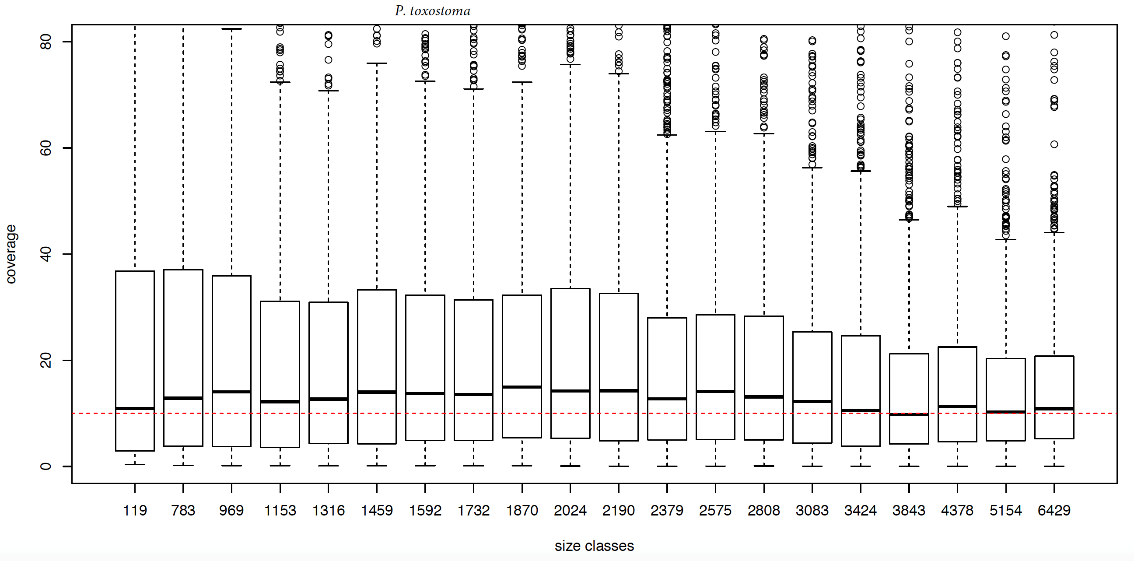


B


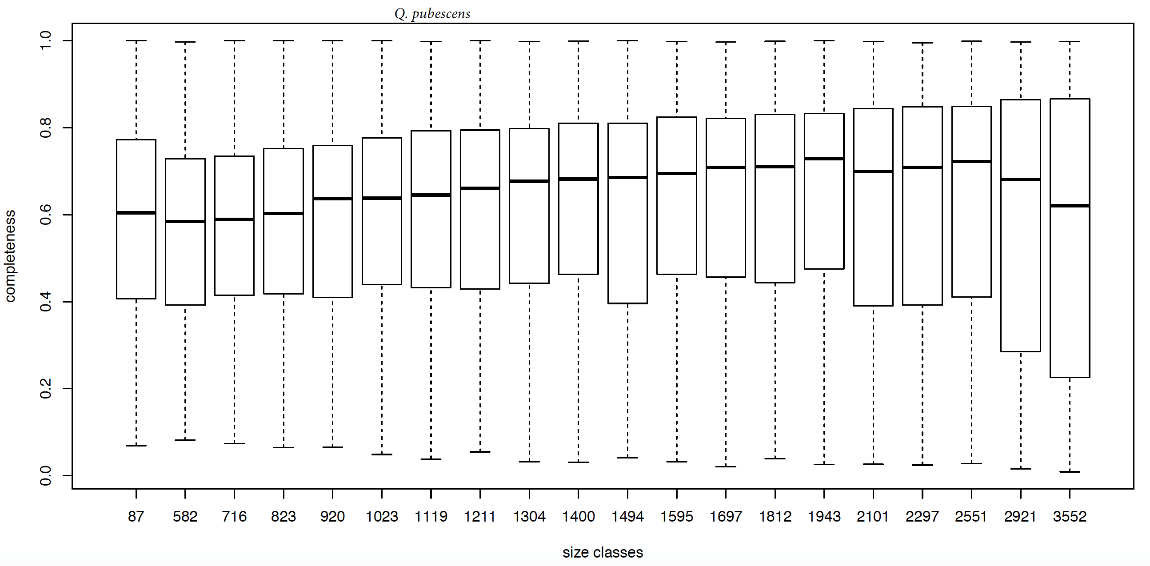


 C


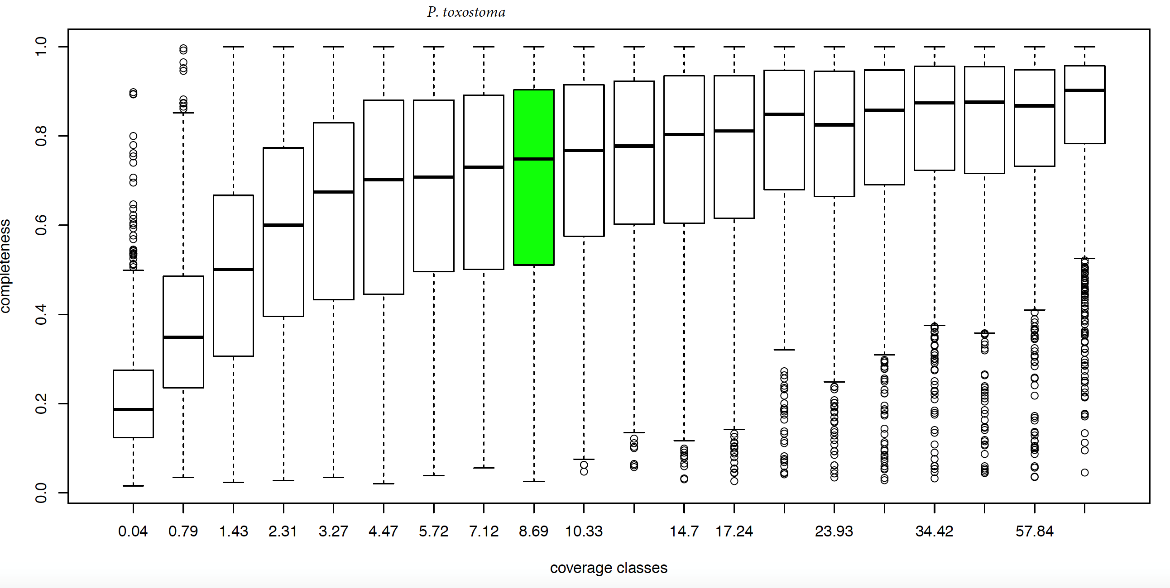

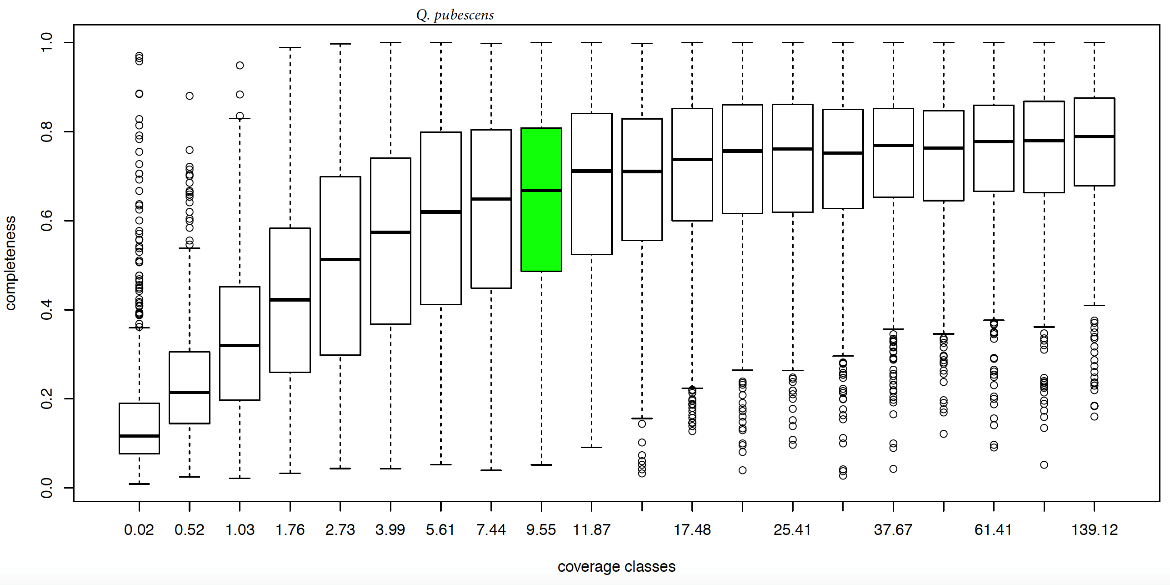


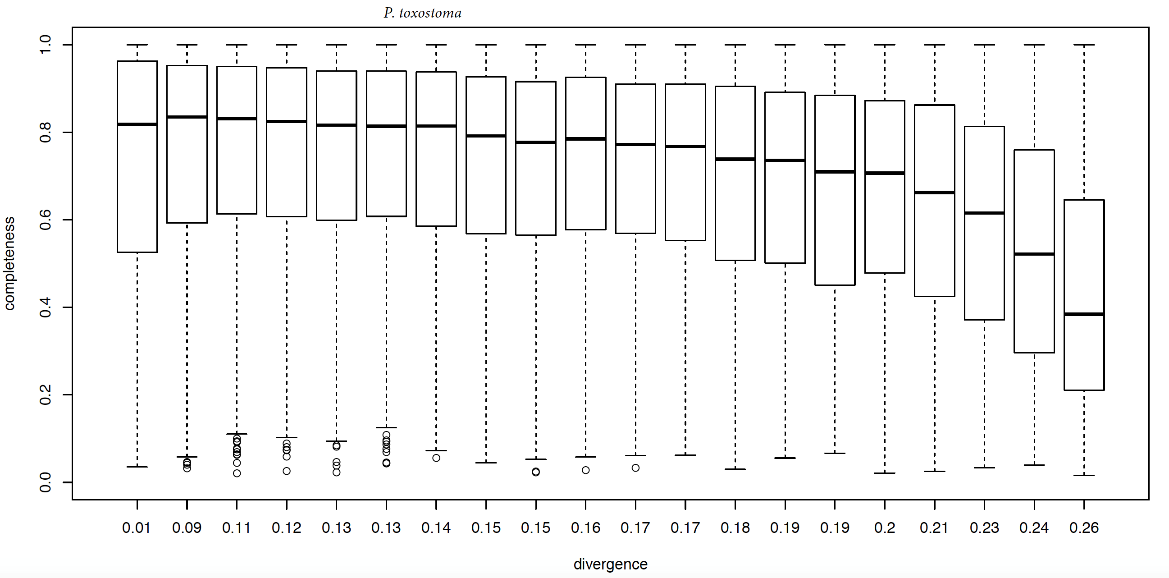
D


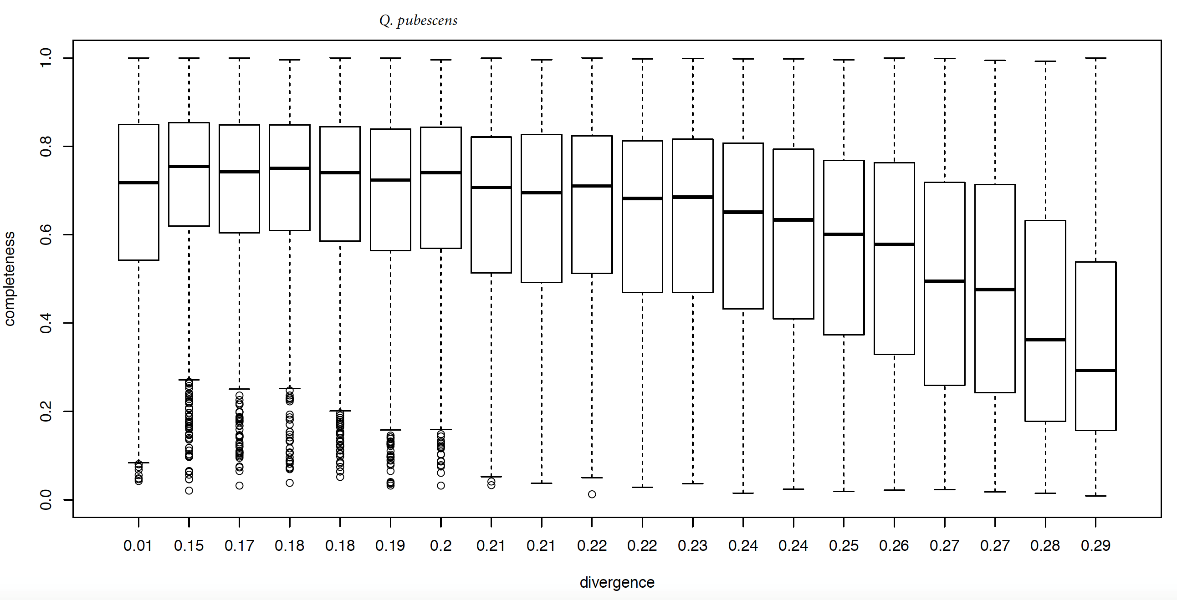

Supplement: S5 Fig — (DOCX) [file pone.0185020.s012.docx]
